# Relationship Between Periodontal Disease and Serum Factors in Patients Undergoing Hemodialysis

**DOI:** 10.2174/1874210601711010701

**Published:** 2017-12-29

**Authors:** Narges Naghsh, Negar Kanuni Sabet, Forozan Vahidi, Ahmad Mogharehabed, Jaber Yaghini

**Affiliations:** 1 Department of Periodontology, Dental Implants Research Center, School of dentistry, Isfahan University of Medical Sciences, Isfahan, Iran; 2 Department of Periodontology, Torabinejad research center, School of dentistry, Isfahan University of Medical Sciences, Isfahan, Iran

**Keywords:** Periodontal disease, Dialysis, Serum factors, Cystatin C, Clinical attachment loss (CAL), Gingival index (GI)

## Abstract

**Background:**

Chronic renal disease is a common condition with several recognized risk factors. Periodontal disease is a recently suggested risk factor for renal disease. We aimed to assess the relationship between periodontal disease and several serum factors in patients undergoing hemodialysis.

**Methods:**

This descriptive cross-sectional study was conducted on 57 patients undergoing hemodialysis. Periodontal examination was done by measuring the mean Pocket Depth (PD), Silness-Löe Plaque Index (PI), Ainamo and Bay Bleeding On Probing (BOP), Löe and Silness Gingival Index (GI) and Clinical Attachment Loss (CAL). Serum levels of albumin, calcium, phosphorus, hemoglobin, ferritin and creatinine were measured *via* a routine blood test. Cystatin C was separately measured. Data were analyzed using independent t-test, Pearson’s correlation coefficient, chi square test and Mann Whitney test (alpha=0.05).

**Results:**

37 men and 20 women were evaluated. Of these, 26.3% had periodontitis and 73.7% had gingivitis. Serum level of albumin (*P*=0.02) and ferritin (*P*=0.043) in patients with periodontitis was significantly higher than that in patients with gingivitis. The serum level of creatinine (*P*=0.02), cystatin C (*P*=0.013), calcium (*P*=0.046) and phosphorus (*P*=0.037) had a significant correlation with severity of periodontitis and increase in CAL.

**Conclusion:**

Increase in the serum levels of albumin and ferritin was related to the progression of gingivitis to periodontitis. Also, the serum levels of creatinine, cystatin C, calcium and phosphorus increased with an increase in CAL.

## 1 INTRODUCTION

Chronic renal disease, diabetes mellitus and cardiovascular diseases are non-contagious, non-communicable diseases [1]. These conditions have several recognized risk factors such as obesity, hypertension and hypercholesterolemia [2]. Periodontal disease is a recently suggested risk factor, which is caused by gram-negative bacteria present in dental plaque [3]. Systemic diseases affect the oral environment by compromising the immune system, causing oral manifestations, impairing collagen synthesis, and creating inflammation as in patients with diabetes [4]. On the other hand, periopathogenic bacteria in active phases of disease produce inflammatory cytokines, which trigger local and systemic inflammatory responses, cause endothelial dysfunction and increase the risk of cardiovascular disease and atherosclerosis [5, 6].

Ioannidou and colleagues [7] evaluated the effect of periodontal disease on systemic inflammation by measuring C Reactive Protein (CRP) as a factor indicative of systemic inflammation in patients with chronic renal disease. They found that patients with periodontitis had higher levels of CRP than patients without periodontitis. However, it seems that the serum level of albumin is a more important factor than CRP for determining the prognosis of chronic renal disease and quality of hemodialysis. Consistently, evidence shows that factors such as BMI affect the relationship between CRP and periodontitis [8, 9].

Increased incidence of secondary hyperparathyroidism and 1/25-dihydroxy vitamin D3 deficiency are two important side effects of chronic renal disease and both result in a reduction in bone density and increase in bone loss, which increases serum levels of calcium and phosphorus in these patients [10]. On the other hand, considering the reduction in erythropoietin in renal insufficiency and its physiological role in increased production of red blood cells in bone marrow, reduction in hemoglobin and ferritin level is expected in case of renal insufficiency [11].

Serum creatinine is a commonly used marker for the assessment of Glomerular Filtration Rate (GFR) and renal function. Its serum level increases in patients with chronic renal disease due to decreased renal clearance [12]. Serum creatinine is influenced by factors such as muscle mass index [13]. Cystatin C is a regulator of endogenous cysteine protease, which is expressed and secreted by all nucleated cells [14]. This factor has recently gained increasing attention. Cystatin C is a low-weight molecule, which is completely filtered in the glomeruli and is reabsorbed by epithelial cells in the proximal tube and catabolized. Thus, it does not return to blood circulation. Also, evidence shows that cystatin C has a closer association with GFR than creatinine and its plasma level is not influenced by age, sex, or muscle mass [15, 16]. Cystatin C is an anti-inflammatory factor and decreases the secretion of cathepsin K, which is a cysteine protease that plays an important role in bone loss especially in the alveolar bone [17]. Some studies have shown that salivary level of cystatin C in patients with periodontitis is lower than that in healthy individuals [18] while some studies have shown an increase in salivary and serum level of cystatin C in patients with periodontitis and have reported its reduction following periodontal therapy [19].

Considering the role of periodontal disease in the development of systemic inflammation and efficacy of some serum factors as markers of renal function, we aimed to assess the relationship between periodontal disease and serum albumin, calcium, phosphorus, ferritin, hemoglobin, creatinine, and Cystatin C in patients undergoing hemodialysis.

## 2 MATERIALS AND METHODS

In this descriptive, cross-sectional study, 57 patients undergoing hemodialysis (d=0.26s, where s is the standard deviation of each variable) referring to Shariati and Hojatiyeh Hospitals in Isfahan were evaluated. The inclusion criteria were age over 18 years, signing inform consent form, having a minimum of six teeth, having symptoms of periodontal inflammation and a minimum of six months passed since their hemodialysis. Considering the significant effect of thyroid disorders, tumors and Alzheimer’s disease on serum level of cystatin C, patients with these conditions were excluded. Patients with invasive periodontitis, significant clinical infections, diabetes mellitus, rheumatoid arthritis (due to its effect on bone metabolism) and patients with a history of taking bisphosphonates, steroids or contraceptives, those who took antibiotics or anti-inflammatory drugs in the past three months and pregnant or nursing women were also excluded from the study. The study protocol was approved by Ethics Committee of Isfahan University of Medical Sciences.

Patients were briefed about the study and signed informed consent forms. Demographic information of patients including age, sex, level of education, socioeconomic status, cigarette smoking, and duration of hemodialysis were recorded. Intraoral clinical examination included measurement of the mean Pocket Depth (PD), Silness-Löe Plaque Index (PI) [20], Ainamo and Bay Bleeding On Probing (BOP) [21], Löe and Silness Gingival Index (GI) [22] and Clinical Attachment Loss (CAL) in all teeth except for third molars. For BOP assessment, a periodontal probe was used for gentle probing of four sites around each tooth; 30-60 seconds later, the presence/absence of bleeding was recorded and the percentage of bleeding sites was calculated. PD was determined by measuring the distance between the gingival margin and PD [23] at mesial, distal, buccal, and lingual sites using a Williams probe (Towne, Pakistan) and the mean PD was calculated. The PI was measured in mesial, distal, buccal, and lingual sites of each tooth except for third molars. According to the amount of plaque, each site was allocated a score from 0 to 3 as follows: 0=No plaque, 1=one layer of plaque adhered to free gingival margin and adjacent areas, which may be detectable by a probe, 2=accumulation of soft debris in gingival sulcus, on tooth surface or gingival margin noticeable by naked eye, and 3=Abundant plaque in gingival sulcus, gingival margin and tooth surfaces. The four numbers for each tooth were added and divided by 4 and PI for each tooth was calculated as such. The mean PI for each patient was then calculated.

Gingival index was measured by assessing the gingival inflammation in mesial, distal, buccal, and lingual sites of each tooth except for third molars. Based on the degree of inflammation, each site was allocated a score from 0 to 3 as follows: 0=Normal gingiva without inflammation, 1=mild inflammation, slight change in gingival color, slight edema, and no BOP, 2=moderate inflammation, edema, and BOP, and 3=severe inflammation, edema, redness, and spontaneous bleeding. The four values were added and divided by 4 to obtain the GI for each tooth. The mean GI for each patient was also calculated.

Periodontitis was diagnosed according to the criteria by the American Academy of Periodontology: a minimum of two sites with CAL ≥ 4mm at the interproximal area or minimum of 2 interproximal areas with PD ≥ 5mm in different teeth [24]. Also, CAL was calculated by measuring the distance from the cementoenamel junction to PD [23] using a Williams probe in teeth with CAL and the mean value for each patient was calculated.

Serum factors except for Cystatin C were measured *via* a routine blood test. The mean time interval between clinical examination of patients and their routine blood test did not exceed two weeks. To measure the level of Cystatin C, blood samples were drawn before dialysis. Blood samples were transferred to a laboratory and within a maximum of 20 minutes, blood serum was separated and frozen at -20°C. Serum level of Cystatin C was measured in Shariati Hospital using Eastbiopharm Human Cystatin C ELISA Kit (Hangzhou, China). Data were analyzed using SPSS software, version 20. Independent t, Pearson’s correlation coefficient, Chi-square, and Mann-Whitney tests were used as appropriated (α=0.05).

## 3 RESULTS

In this study, 57 patients (37 men and 20 women) undergoing hemodialysis with a mean±SD age of 47.9±14.6 years (range: 21-84 years) were enrolled. The mean±SD duration of hemodialysis was 48.7±47.5 months. 59.6% of the patients had a level of education below high school diploma, 33.3% had high school diploma, and 7% had level of education higher than high school diploma. Socioeconomic status was good in 35.1%, moderate in 61.4% and poor in 3.5% of the patients. Also, of the 57 patients, 5 (8.8%) were smokers, 15 (26.3%) had periodontitis, and 42 (73.7%) had gingivitis (mild type of periodontal disease associated with gingival inflammation, tooth supporting structures are not involved and it is reversible [25]).

The demographic information of the patients with gingivitis and periodontitis was analyzed using Chi-square, independent t, and Mann-Whitney tests. Except for age (*P*=0.001), the two groups (gingivitis/periodontitis) were not significantly different in terms of other demographic parameters. Independent t test showed that the mean age in patients with periodontitis was significantly higher than those with gingivitis (*P*=0.001). Independent t test showed that the mean PD (*P*<0.001), PI (*P*=0.005), GI (*P*=0.001), BOP (*P*=0.008), and CAL (*P*<0.001) were significantly greater in patients with periodontitis than those with gingivitis (Table **1**).

Moreover, the mean serum levels of albumin (*P*=0.02) and ferritin (*P*=0.043) were significantly lower in patients with periodontitis than those without periodontitis (Table **2**). Pearson’s correlation coefficient showed that CAL, which indicates the severity of periodontitis, had a significant association with serum level of Calcium (*P*=0.046, r=0.45), Phosphorus (*P*=0.037, r=0.47), Creatinine (*P*=0.02, r=0.6) and Cystatin C (*P*=0.013, r=0.62) in patients with periodontitis (Table **3**).

## 4 DISCUSSION

It has been generally accepted that periodontitis could be an important risk factor for CKD and kidney failure [26] based on the following two points: (a) systemic inflammation is affected by periodontal inflammation and (b) periodontal bacteria and/or their products can enter the bloodstream [27]. In addition, the oral health of patients undergoing dialysis therapy is frequently found to be poor due to insufficient oral hygiene caused by their poor condition.

This study, evaluated the correlation of periodontal disease with serum factors such as albumin, calcium, phosphorus, hemoglobin, ferritin, creatinine and Cystatin C (which are all related to renal function) in patients undergoing hemodialysis. The results showed that serum level of albumin had a significant inverse correlation with the mean PD. Also, a significant inverse correlation was noted between serum ferritin and PI and GI. Based on the results, serum levels of Creatinine, Cystatin C, Calcium, and Phosphorus were significantly correlated with CAL.

The results showed that serum levels of albumin and ferritin in patients with periodontitis were significantly lower than those with gingivitis.

Several studies have attempted to demonstrate a relationship between periodontal disease and renal disease, but the results were inconsistent [28].

Reduction in serum albumin in patients with chronic renal disease increases the risk of morbidity and mortality in these patients [29]. Albumin is an acute phase protein. Its serum concentration decreases in systemic inflammatory conditions [30]. The process of inflammation and subsequent production of inflammatory cytokines can decrease protein synthesis in the liver and since albumin is the most abundant serum protein produced by the liver, its serum level often decreases in inflammatory conditions [31]. In patients with periodontitis, because of damage to sulcular epithelium (which serves as a barrier against microorganisms), bacteria enter into the blood stream and cause systemic inflammation [32], which also explains reduction in serum albumin in these patients. In our study, periodontal parameters were significantly higher in periodontitis patients compared to those gingivitis, and serum level of albumin had a significant inverse correlation with the mean PD.

In two studies [8, 33] serum level of albumin in renal patients with severe periodontal disease was lower than that in patients with milder form of periodontal disease that were in agreement with our study.

Iron insufficiency in patients undergoing hemodialysis is a common finding. Serum level of ferritin is significantly correlated with bone marrow iron content in these patients. Thus, serum ferritin is a commonly used marker for assessment of iron deficiency in patients undergoing hemodialysis [34]. Also, ferritin is an acute phase reactant and its serum level increases in systemic inflammation [35]. However, some studies have reported that the effect of inflammation on serum level of ferritin in patients undergoing hemodialysis depends on the iron content of the body and inflammation affects serum ferritin only when iron content is sufficient. In other words, if the iron content and serum level of ferritin are low, ferritin concentration and inflammation are not correlated [36]. Moreover, inflammatory cytokines such as IL-6, IL-1α, and TNF-α released following systemic inflammation are also synthesized due to periodontal disease; these mediators inhibit the formation of red blood cells in the bone marrow and cause anemia [37]. Studies on the relationship of serum level of ferritin and periodontal disease in patients undergoing hemodialysis are limited. In our study, serum level of ferritin in patients undergoing hemodialysis with periodontitis was significantly lower than that in patients with gingivitis. Also, serum level of ferritin had a significant inverse correlation with PI and GI. Rodrigues and colleagues [5] found no significant association between serum level of ferritin and periodontal disease although the mean serum level of ferritin was lower in periodontitis patients. Further studies are required on this topic.

The results of our study showed that a significant association existed between serum level of creatinine and Cystatin C in patients undergoing hemodialysis and CAL. In a study by Kshirsagar and co-workers [36] patients with periodontitis had lower GFR and higher serum level of creatinine compared to healthy individuals .Serum level of creatinine is a commonly used marker for assessment of GFR and it also serves as an index for assessment of renal function. In chronic renal disease, as a result of decreased renal clearance, serum level of creatinine increases [12]. However, serum creatinine is also affected by factors such as muscle mass index [13]. Cystatin C is another marker for assessment of GFR and has fewer innate limitations than creatinine. Its serum level increases earlier than that of creatinine in progressive chronic renal disease [38]. Periodontitis can compromise renal function in several ways [39]. Generally, patients with periodontitis have low-intensity recurrent and chronic bacteremia [40]. By aggravation of periodontal disease and damage to sulcular epithelium, bacteria enter the bloodstream [32]. Bacterial antigens trigger immune host response. Polysaccharide cell wall of bacteria can induce the release of inflammatory mediators such as IL-6, prostaglandin E2, and thromboxane B2. These factors can expedite angiogenesis, thrombus formation and platelet aggregation *via* several mechanisms. Moreover, thromboxane causes vasoconstriction, and its chronic release can cause a chronic reduction in renal blood flow. Atherogenesis of renal artery and arterioles also causes ischemia, glomerular sclerosis, and severe renal insufficiency. Furthermore, periodontal pathogens can directly reach the renal nephrons *via* the bloodstream and invade the endothelium of capillaries and mesenchymal cells and damage the nephrons [39].

Thus it can be possible that periodontal disease aggravates renal insufficiency and decreases GFR and this leads to increased in serum level of creatinine and Cystatine C.

In addition to the role of cystatin C as a marker of renal function, this factor inhibits the activity of cystatin protease, which plays an important role as an anti-inflammatory factor [41]. Cathepsin K is a cystatin protease mainly expressed by osteoclasts and plays an important role in alveolar bone resorption. The level of this enzyme increases in patients with periodontitis, and it is known as a marker for the assessment of the activity of osteoclasts in periodontal disease [42]. By an increase in the level of Cathepsin K, level of Cystatin C as a marker regulating the activity of this enzyme increases [17]. Our study showed that the level of Cystatin C increased with an increase in CAL. In the study by Sharma and colleagues [19], salivary and serum level of Cystatin C increased with an increase in severity of periodontal disease.

In our study, serum level of calcium and phosphorus had a significant association with CAL. By an increase in severity of periodontitis, serum levels of calcium and phosphorus increased in patients undergoing hemodialysis. Increase in serum level of calcium and phosphorus in patients undergoing hemodialysis is a strong risk factor for morbidity and mortality [43]. High serum level of phosphorus in patients with chronic renal disease causes vascular calcification in these patients and increases the risk of cardiovascular disease. High serum level of phosphorus can result in differentiation of smooth muscle cells in vessel walls to osteoblasts, which results in formation of atheromatous plaque [44]. Kidneys play a role in regulating serum levels of calcium and phosphorus [45]. As stated earlier, inflammation following aggravation of periodontal disease causes progressive renal damage and compromises its function [39]. Thus, it may be stated that due to decreased renal function as a result of periodontal disease and role of kidneys in regulating serum calcium and phosphorus, these values increase with the aggravation of periodontitis. The relationship between periodontal disease and chronic renal disease is mutual, such that chronic renal disease has oral manifestations and periodontal disease affects the function of kidneys. Increased secretion of parathyroid hormone in patients with chronic renal disease due to secondary hyperparathyroidism can result in bone calcium loss and jawbone abnormalities. Also, these patients have higher dental calculus because of increased serum level of calcium and phosphorus as a result of secondary hyperparathyroidism, which compromises periodontal health [46]. Rodrigues and colleagues [5] reported that serum level of calcium has no significant association with periodontal disease in patients undergoing hemodialysis. Also, they showed that periodontitis decreases the serum level of phosphorus in these patients. Further investigations on this association seem imperative.

Considering the association of periodontal disease with some serum factors in hemodialysis patients, it appears that periodontal therapy and oral health promotion in these patients can help control the serum level of inflammatory factors. One limitation of this study was its cross-sectional design. Clinical trials are required to assess the serum level of these factors before and after periodontal therapy to reach more definite results and compare the patients undergoing hemodialysis and normal patients without hemodialysis. Clinical examination of patients undergoing hemodialysis was difficult and the patients often had poor cooperation. Also, the number of patients undergoing hemodialysis with adequate number of teeth for inclusion in our study was limited and out of 220 examined patients, only 70 were dentate and 57 met our inclusion criteria.

## CONCLUSION

Our results showed that severity of periodontal disease was correlated with serum level of some factors in patients undergoing hemodialysis. Since periodontal factors evaluated in our study were significantly higher in patients with periodontitis compared with those with gingivitis, it may be concluded periodontal disease that is correlated with the reduction of serum levels of albumin and ferritin in these patients. Also, by an increase in CAL, serum levels of Creatinine, Cystatin C, Calcium, and Phosphorous increased.

## Figures and Tables

**Table 1:** The mean, standard deviation, minimum and maximum of periodontal parameters in patients undergoing hemodialysis with gingivitis and periodontitis.

Variable	Gingivitis				Periodontitis				*P* value
	Mean	SD	Min.	Max.	Mean	SD	Min.	Max.	
Pocket depth	1.51	0.22	1.05	2.13	1.96	0.33	1.26	2.54	<0.001
Plaque index	1.55	0.48	0.65	2.75	1.97	0.42	1.37	3	0.005
Gingival index	1.25	0.41	0.33	1.95	1.64	0.23	1.3	2.25	0.001
Bleeding on probing	32.64	17.3	5	70	46.4	14.1	31	79	0.008
Clinical attachment loss	0	0	0	0	4.33	0.69	3	6	<0.001

**Table 2:** The mean and standard deviation of serum markers in patients undergoing hemodialysis with gingivitis and periodontitis.

Variable	Periodontitis		Gingivitis		*P* value
	Mean	SD	Mean	SD	
Albumin (g/dL)	3.83	0.32	4.14	0.54	0.02
Calcium (mg/dL)	8.41	0.92	8.89	0.77	0.086
Phosphorus (mg/dL)	5.46	1.39	5.82	1.52	0.43
Hemoglobin (g/dL)	11.77	2.79	11.53	1.72	0.75
Ferritin (ng/mL)	378.07	87.38	577.63	60.54	0.043
Creatinine (mg/dL)	7.54	1.75	8.52	1.96	0.096
Cystatin C (ng/mL)	56.73	20.95	54.27	19.89	0.69

**Table 3:** Pearson’s correlation coefficient for the association between serum markers and periodontal parameters in patients with hemodialysis.

Variable	PD		PI		GI		BOP		CAL	
	r	P value	r	P value	r	P value	r	P value	R	P value
Albumin (g/dL)	-0.26	0.025	-0.20	0.07	-0.194	0.07	-0.163	0.11	-0.002	0.99
Calcium (mg/dL)	-0.128	0.17	-0.016	0.90	0.076	0.57	-0.073	0.59	0.45	0.046
Phosphorus (mg/dL)	-0.01	0.94	-0.19	0.076	-0.134	0.16	-0.165	0.11	0.47	0.037
Hemoglobin (g/dL)	0.03	0.98	-0.031	0.82	0.004	0.97	0.014	0.91	0.01	0.97
Ferritin (ng/mL)	-0.14	0.15	-0.277	0.02	-0.236	0.04	-0.144	0.14	-0.128	0.65
Creatinine (mg/dL)	-0.207	0.06	-0.087	0.52	-0.135	0.16	-0.038	0.77	0.6	0.019
Cystatin C (ng/mL)	0.13	0.17	-0.01	0.94	0.195	0.07	-0.176	0.095	0.62	0.013
